# GSK3β Overexpression Indicates Poor Prognosis and Its Inhibition Reduces Cell Proliferation and Survival of Non-Small Cell Lung Cancer Cells

**DOI:** 10.1371/journal.pone.0091231

**Published:** 2014-03-11

**Authors:** Jing Zeng, Dan Liu, Zhixin Qiu, Yi Huang, Bojiang Chen, Lei Wang, Huan Xu, Na Huang, Lunxu Liu, Weimin Li

**Affiliations:** 1 Department of Respiratory Medicine, West China Hospital, Sichuan University, Chengdu City, Sichuan Province, China; 2 Clinical Laboratory Department, Sichuan Academy of Medical Sciences & Sichuan Provincial People’s Hospital, Chengdu City, Sichuan Province, China; 3 Department of Pathology, West China Hospital, Sichuan University, West China Hospital, Sichuan University, Chengdu City, Sichuan Province, China; 4 Thoracic and Cardiovascular Surgery, West China Hospital, Sichuan University, Chengdu City, Sichuan Province, China; H. Lee Moffitt Cancer Center & Research Institute, United States of America

## Abstract

**Background:**

Glycogen synthase kinase 3 beta (GSK3β) is centrally involved in diverse cellular processes, including proliferation and apoptosis. This study aimed to investigate the influence of GSK3β expression on the prognosis of human non-small cell lung cancer (NSCLC) and the effects of GSK3β inhibition in NSCLC cell lines.

**Methods:**

Immunohistochemical and western blot assays were used to evaluate the GSK3β expression level in human NSCLC tissues. Lentiviral RNA interference was performed to inhibit the expression of GSK3β in the A549, H292, H1299 and SK-MES-1 cell lines. Cell survival, apoptosis and motility were evaluated *in vivo* and *in vitro*.

**Results:**

The levels of GSK3β were greater in NSCLC tissues (n = 211) than in control tissues (n = 194) (*P*<0.001). The 5-year follow-up analysis showed that positive GSK3β expression was indicative of poor prognosis (*P* = 0.006). Furthermore, knockdown of GSK3β in NSCLC cell lines suppressed cell proliferation, arrested tumor cells in G0/G1 phase, induced apoptosis and reduced cell motility. A xenograft model showed that the deregulation of GSK3β attenuated tumorigenesis, as confirmed by reduced cell proliferation based on Ki-67 and significantly increased apoptotic cell death. The inhibition of GSK3β had inconsistent effects on the expression of β-catenin, depending on the cell type examined.

**Conclusion:**

Aberrant expression of GSK3β serves as an independent marker of poor prognosis for NSCLC. The inhibition of GSK3β suppressed tumorigenesis by attenuating cell proliferation, increasing apoptosis and restraining cell motility. These results identify GSK3β as a tumor promoter and a potential therapeutic target in NSCLC.

## Introduction

Lung cancer has been the leading cause of worldwide cancer-related deaths for decades [1]. In China, the number of lung cancer cases and deaths related to lung cancer have increased with increasing cigarette abuse and pollution levels [2]. Non-small cell lung cancer (NSCLC), which primarily includes adenocarcinoma and squamous cell carcinoma, accounts for nearly 85% of lung cancers. Although new therapeutic approaches have been introduced, most patients are still diagnosed at advanced stages, and the 5-year survival rate remains less than 15% [1]. To improve the outcome and quality of life of patients with NSCLC, many recent studies have focused on finding new therapeutic targets and identifying biomarkers to facilitate individualized treatment [3].

Glycogen synthase kinase 3 beta (GSK3β) is a multifunctional serine/threonine protein kinase that was originally isolated from rabbit skeletal muscle [4]. Under resting conditions, GSK3β is constitutively active due to tyrosine-216 phosphorylation, and it phosphorylates and inhibits a diverse group of pro-oncogenic substrates, such as β-catenin, cyclin D1, c-Jun, c-Myc and CREB. In turn, the phosphorylation of serine-9 inactivates GSK3β [5]–[7]. Based on these functions, GSK3β is a potential tumor suppressor, and the inactivation of GSK3β has been reported in many cancer cells [8], [9]. However, many studies have demonstrated that GSK3β can positively regulate the proliferation and apoptosis of tumor cells [10]–[18]. In addition, studies have shown that the inhibition of GSK3β attenuates survival and proliferation and induces apoptosis in various types of cancers, such as pancreatic cancer [11], colorectal cancer [12], [15] and bladder cancer [18]. However, the role of GSK3β differs in different cancers [19], and the precise role of GSK3β in NSCLC remains unclear.

In this study, we explored the expression of GSK3β in human NSCLC tissues and its prognostic significance. In the H292, H1299, SK-MES-1 and A549 NSCLC cell lines, GSK3β expression was inhibited by small hairpin RNA(shRNA) transferred by lentiviral vector. Stable knockdown cell lines were verified and used in further research. Tumorigenesis, proliferation, apoptosis and cell migration were also assessed both *in vivo* and *in vitro*, and the results demonstrated that aberrant expression of GSK3β served as an independent indicator of poor prognosis for NSCLC. Moreover, the inhibition of GSK3β by RNA interference suppressed tumorigenesis by attenuating cell proliferation, increasing apoptosis and suppressing cell invasion. Together, these findings identify GSK3β as a tumor promoter and a potential therapeutic target for NSCLC.

## Materials and Methods

### 1. Patients and tissue collection

Surgically resected NSCLC tissues (n = 211) and specimens of normal lung adjacent to the tumor tissues (n = 194) were obtained from West China Hospital, Sichuan University. None of the patients had been treated with any preoperative chemotherapy or radiotherapy. Follow-up information was available for 160 cases. The pathologic stages were determined according to the International Union Against Cancer (UICC) tumor-node-metastasis (TNM) classification system for malignant tumors. To further explore, the extent of differentiation and histological type was determined according to the World Health Organization classification for NSCLC. After surgical resection, all patients received standard therapies according to the 2004 NCCN Clinical Practice Guidelines in Oncology for NSCLC. Written informed consent was obtained from each patient, and institutional review board approval of this study was obtained from West China Hospital, Sichuan University.

### 2. Stable gene silencing using small interfering RNA mediated by lentiviral vector

The human lung NSCLC cell lines A549, H292, H1299 and SK-MES-1, which were obtained from the American Type Culture Collection (ATCC), were cultured according to the ATCC protocols.

Sequences (GAAGAAAGATGAGGTCTAT) targeting GSK3β (GenBank accession: NM_002093) that were designed using the BLOCK-iT RNA interference (RNAi) Designer (Invitrogen, Carlsbad, CA) were selected. A sequence unrelated to the GSK3β gene (TTCTCCGAACGTGTCACGT) was designed as a negative control (NC) [20]. The pGCSIL-GFP lentiviral vector was purchased from GeneChem and NeuronBiotech Corp (Shanghai, China). Green fluorescent protein (GFP) was used as marker to observe and sort these transfected tumor cells with fluorescence-activated cell sorting (FACS). The effects of RNA interference were assessed by RT-PCR (at 5^th^ days after transfection) and western blotting (at 7^th^ days after transfection). Then, NSCLC cell lines with stable and sustained GSK3β gene knock down were obtained for further *in vivo* and *in vitro* studies. The cells infected with the lentivirus that delivered the sequence targeting GSK3 were named as the knock down group (KD group). Similarly, cells containing the lentivirus-delivered the NC sequence were named as the NC group; and these tumor cells did not infected by lentivirus were used as a normal control and named as the control group (CON group).

### 3. Tumorigenesis in xenografted mice

Nude mice (BALB/c-nu/nu, n = 6 for each group, equal numbers of males and females, 6–8 weeks old) were supplied by the Laboratory Animal Center of Sichuan University. The mice were housed in laminar flow cabinets under specific pathogen-free conditions and fed ad libitum. All studies involving mice were conducted according to the National Institutes of Health Guidelines for the Care and Use of Laboratory Animals. Approval for this study was given by the Institutional Animal Care and Treatment Committee of Sichuan University.

Following treatment with different viruses, exponentially growing A549 cells were subcutaneously injected into the backs of Balb/c nude mice (1×10^6^/ml each). The tumor volumes were assessed every 3 days according to the following formula: tumor volume (mm^3^)  =  d^2^×D×0.52. Four weeks after tumor implantation, the mice were painlessly sacrificed. Their organs were examined for gross evidence of anatomical changes.

### 4. Cell proliferation assays

The Cell Counting Kit-8 (CCK-8; Dojindo, Rockville, USA) was used to assess cell proliferation according to the manufacturer's protocol. Tumor cells (2×10^3^ per well) were seeded in 96-well culture plates, and treated with 10% FBS and incubated at 37°C. The optical density at 450 nm was measured at 24, 48, 72, 96 and 120 h after virus transfection. The data shown are representative of 3 independent experiments and are presented as the mean ± S.D.

### 5. Cell cycle analysis

Seventy-two hours after transfection, cell cycle data were obtained by analyzing of PI-stained cells using fluorescence-activated cell sorting (FACS) with a FACSCalibur flow cytometer (Becton Dickinson, Franklin Lakes, USA). For each sample, at least 3×10^5^ cells were counted, and the data were analyzed with BD CellQuest software. The data shown are representative of 3 independent experiments and are presented as the mean ± S.D.

### 6. Apoptosis analysis

Tumor cells (approximately 5×10^5^) were stained with 5 µl of Annexin V-APC and 7AAD (KeyGen, Nanjing, China) at room temperature and then analyzed by flow cytometry within 1 h. The Annexin V(+)/7AAD(–) cells were regarded as apoptotic cells.

The TUNEL method (In Situ Cell Death Detection Kit AP, Roche, Switzerland) was used to determine the level of apoptosis in xenograft tumor tissues. Apoptotic cells were detected using alkaline phosphatase and stained in red. For each tumor, apoptotic cells in 5 random high-power fields were counted, and the rate of apoptosis was calculated with the following formula:

Apoptosis rate  =  number of apoptotic cells/total number of tumor cells counted × 100%.

### 7. RNA extraction and real-time PCR

The primers for human GSK3β were 5′-ATTTCCAGGGGATAGTGGTGT-3′ (sense) and 5′-TCCTGACGAATCCTTAGTCCAAG-3′ (antisense); and those for GAPDH were 5′-CCATCACCATCTTCCAGG-3′ (sense) and 5′- ATGAGTCCTTCCACGATAC -3′ (antisense). The primers and probes were purchased from GeneChem, Shanghai, China. The mRNA expression levels were quantified in triplicate by real-time RT-PCR using a 2720 thermal cycler (Applied Biosystems, Foster City, California). The relative levels of target transcripts were quantified using the 2(-Delta Delta Ct) method [21] and normalized to the level of human GAPDH transcripts.

### 8. Cell invasion assay

The Cell Invasion Assay Kit (ECM550, Chemicon, California, USA) was used to assess cell invasiveness. After virus transfection, an aliquot of the prepared cell suspension (300 µl, 1.0×10^6^ cells/ml) was added to each upper insert. After 48 h of incubation, the inserts were dipped into staining solution for 20 min to stain the invasive cells on the membrane. Then, the invasive cells in 5 random microscope views were counted. The data shown are representative of 3 independent experiments and are presented as the mean ± S.D.

### 9. Western blotting analysis

Total proteins were extracted from NSCLC tumor tissues and transfected cultured cells and then qualified using a protein extraction kit (KeyGEN, Nanjing, China) and the BCA Protein Assay reagent (Thermo scientific, Rockford, USA). The proteins were separated by SDS–PAGE and visualized by immunoblotting with antibodies specific for GSK3β (#9315, diluted 1:400) and β-catenin (#9582, diluted 1:200) (Cell Signaling Technology, Beverly, USA). After exposure to a chemiluminescent HRP substrate (Millipore, Billerica, USA), the target proteins were detected using a ChemiDoc XRS system (Bio-Rad, Philadelphia, USA), and the images were analyzed with Gel-Pro Analyzer 4.0 software (Media Cybernetics).

### 10. Immunohistochemical assay

GSK3β in NSCLC tumor tissues and the adjacent normal tissues was immunohistochemically stained as described in our previous studies [22]–[24]. Semiquantitative evaluation of the sections was performed by 2 pathologists in a blinded manner. The negative control for immunostaining was performed without primary antibody. Both the fraction and intensity of the immunostained tumor cells were considered. The fraction score was calculated as the average of 5 randomly selected high-power fields assessed by light microscopy as follows: 0, no stained target cells (tumor, bronchial or alveolar cells); 1, <20% of cells stained; 2, 20∼50% of cells stained; and 3, over 50% of cells stained. The intensity score was defined as follows: 0, no appreciable staining of the target cells; 1, barely detectable staining in the cytoplasm and/or nucleus of the target cells; 2, readily apparent brown staining; and 3, dark brown staining. The fraction and intensity scores were multiplied to give a total score ranging from 0 to 9. Scores between 2 and 9 were regarded as positive expression. Immunohistochemical assays were also performed on histological sections of paraffin-embedded xenograft tumors.

Staining of the proliferative marker Ki-67 was performed to assess the growth of the xenograft tumors. The Ki-67 antibody (#MA1-80199, diluted 1:100) was obtained from Thermo Fisher Scientific, Rockford, USA. The Ki-67 labeling index (Ki-67 LI) was calculated according to the following formula:

Ki-67 LI  =  number of Ki-67 positive cells/total number of tumor cells counted × 100%.

### 11. Statistical analysis

SPSS 17.0 for Windows (SPSS Inc., Chicago, Ill, USA) was used for all data analysis. The immunohistochemistry results and their associations with clinical characteristics were analyzed using the chi-square test. Spearman’s rank test was used to analyze the correlation between protein phenotypes. The Kaplan-Meier method was used to analyze univariate survival, and comparisons of the survival distributions among groups were performed using the log-rank test. The prognostic significance of GSK3β expression with respect to other pathological variables was assessed using multivariate Cox regression analysis. The quantitative data are expressed as the mean ± S.D. The statistical significance of differences among the groups was determined by one-way ANOVA followed by Fisher’s PLSD post-hoc test. All *P* values were 2-tailed, and values of *P* < 0.05 were considered statistically significant.

## Results

### 1. GSK3β is upregulated in NSCLC tumor tissues

**Figure 1:**
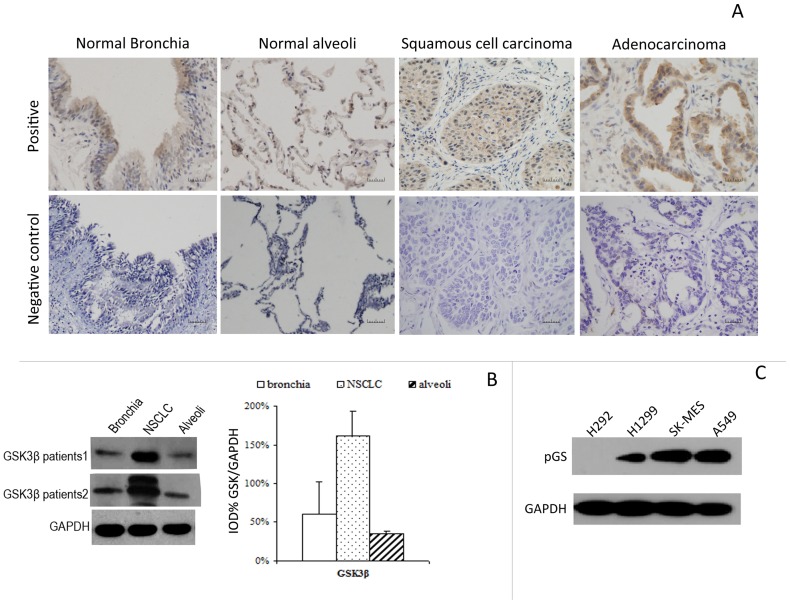
GSK3β is overexpressed and active in NSCLC tumor tissues. (**A**) Typical IHC staining images of human NSCLC tumor and normal lung tissue (magnification × 200). (**B**) Western blotting assays confirmed that in paired normal/tumor tissues from NSCLC cancer patients, higher expression levels of GSK3β in tumors were found compared with their normal counterparts. The experiments were repeated 3 times, and the data are presented as the mean ± SD. *P<0.001. (**C**) Phosphorylated glycogen synthase (pGS), a substrate of GSK3β, was detected in these NSCLC cell lines, except for H292.

Representative images of immunohistochemical staining for GSK3β in NSCLC tissues and the corresponding normal tissues are shown in Figure 1A. GSK3β was prominently expressed in NSCLC (n = 211), primarily in the cytoplasm of tumor cells. Comparisons of the GSK3β protein levels between the tumor and normal lung tissues are shown in Table 1. The results demonstrated that GSK3β level was significantly increased in NSCLC tissues compared with the normal lung tissues (*P*<0.001). Western blotting assays also confirmed that the expression level of GSK3β in tumors was higher than that in their normal counterparts. (*P* = 0.04, Figure 1B).

**Table 1:** The Expressions of GSK3β in NSCLC and Normal Lung Tissue.

Protein	P/N	NSCLC n = 211 (100%)	Normal lung tissue n = 194 (100%)	*P*
GSK3β	P	113 (53.6%)	53(27.3%)	<0.001*
	N	98 (46.4%)	141(72.7%)	

Abbreviations: P, positive; N, negative. * Statistical significant.

As a substrate of GSK3β,the protein expression of phosphorylated glycogen synthase (pGS) was used to indicate the activity of GSK3β. In these 4 cell lines, except in H292 pGS, was detected (Fig 1C), which suggested high activity of GSK3β in human lung cancer.

### 2. Positive expression of GSK3β is associated with poor prognosis for NSCLC

**Figure 2:**
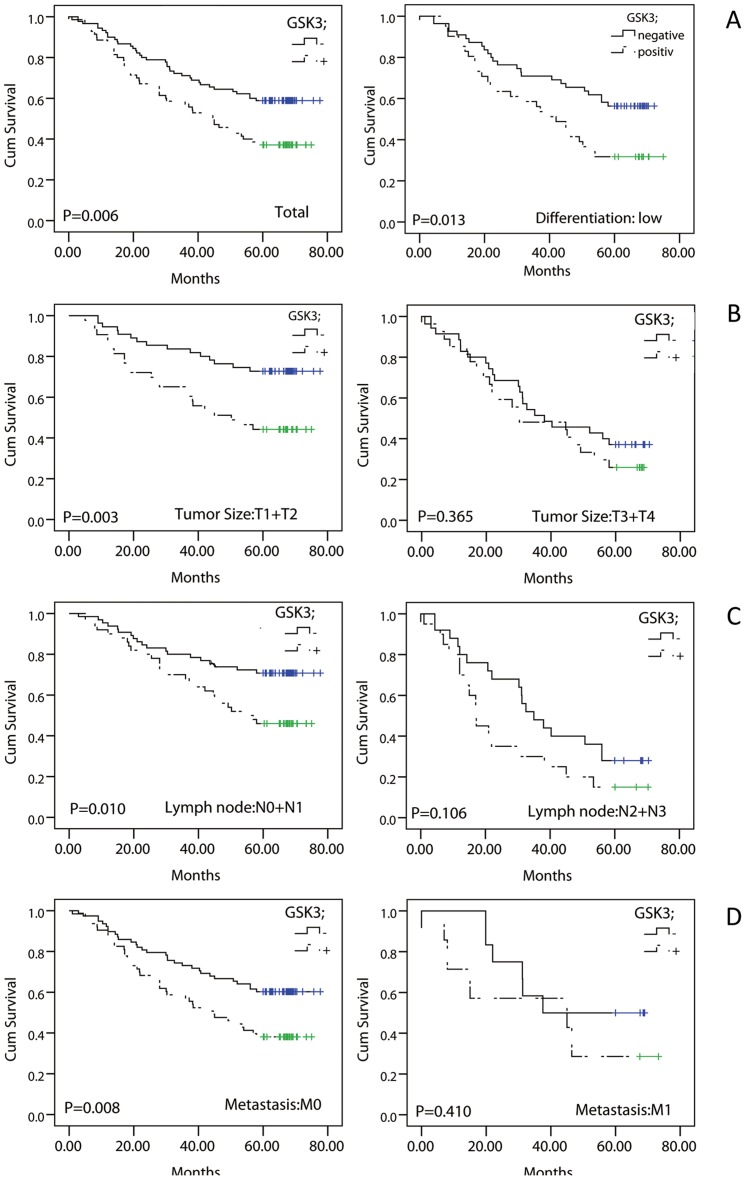
Positive expression of GSK3β is associated with poor prognosis for NSCLC. (**A**) In the entire group, patients with positive GSK3β expression had shorter survival times, *P* = 0.006. Figure 2B-D shows the survival analysis for the subgroups. (**B**) In the lower differentiation subgroup, patients with positive GSK3β expression had significantly shorter survival times, *P* = 0.013. (**C**) In the T1+T2 subgroup, patients with positive GSK3β expression had significantly shorter survival times, *P* = 0.003. (**D**) In the N0+N1 subgroup, patients with positive GSK3β expression had significantly shorter survival times, *P* = 0.010. (E) Patients in the M0 subgroup with positive GSK3β expression had significantly shorter survival times, *P* = 0.008. Kaplan-Meier curves for the investigated proteins were used to compare the positive (*dashed lines*) and negative phenotypes (*solid lines*)**.**

Patients with complete clinical information (160 cases, 39 women and 121 men, 61.19±10.53 years) were enrolled in the survival analysis. The median age was 60.00 years (range, 29 to 83 years). The clinical characteristics and survival analysis results are summarized in Table 2 and Figure 2. The 5-year survival rate for the entire group was 49.07%, and the median follow-up time was 58.00 months (range, 0 to 77.67 months).

**Table 2:** The clinical characteristics of patients, GSK3β expression and the survival analysis.

	GSK3β Expression					Log rank	Median	Log rank	Cox Regression							
Factors	Total		–	+	χ ^2^ *P*	Total	survival	Subgroup	Univariate				Multivariate			
						*P*	(Months)	*P*	HR	95% CI		*P*	HR	95% CI		*P*
**Gender**	Female	39	27	12	.066	.207	56.930	.086								
	Male	121	63	58			60.000	**.** 019*								
**Age**	<60	66	27	39	.337	.409	60.000	.143								
	≥60	94	46	48			50.770	**.** 020*								
**Histological type**	A	80	48	32	.748	.305	38.000	.111								
	SqCC	51	26	25			62.000	**.** 002*								
	Other	29	16	13			60.000	.471								
**Differentiation**	Low	96	55	41	.748	.282	53.430	**.** 013*								
	Moderate/high	64	35	29			60.000	.141								
**Tumor size**	T1+T2	98	55	43	1.000	**.** 000*	62.170	**.** 003*	2.136	1.380 – 3.308		**.** 001*	1.651	1.006 – 2.710		**.** 047*
	T3+T4	62	35	27			35.000	.365								
**Lymph node**	N0+N1	115	65	50	1.000	**.** 000*	62.170	**.** 010*	2.957	1.898 – 4.608		**.** 000*	2.655	1.414 – 4.985		**.** 002*
	N2+N3	45	25	20			30.370	.106								
**Distant metastasis**	M0	141	78	63	.625	**.778**	60.000	**.** 008*	1.292	0.684 – 2.439		**0.43**				
	M1	19	12	7			45.000	.410								
**Clinical stages**	I+II	84	46	38	.751	**.** 001*	62.600	**.** 045*	2.137	1.368 – 3.340		**.** 001*	0.981	0.494 – 1.945		**0.955**
	III+IV	76	44	32			38.230	**.** 024*								
**GSK3β**	N	90				**.** 006*	60.000		1.837	1.185 – 2.846		**.** 007*	1.947	1.252 – 3.026		**.** 003*
	P	70					44.600									

Abbreviations: HR hazard ratio; CI confidence interval. * Statistical significant; N, negative, P, positive; A, adenocarcinoma, SqCC, squamous cell cancer.

Log rank analysis for total group is the survival effect of various clinical parameters on whole group, Log rank analysis for subgroup is the survival effect of GSK3β positive expression on different subgroup.

Univariate analysis was performed to estimate the relation between clinical characteristics and GSK3β expression. As shown in Table 2, NSCLC patients with different clinical characteristics, such as sex, age, histological type and tumor-node-metastasis (TNM) stage, demonstrated similar levels of GSK3β. The prognostic significance of positive GSK3β expression in different subgroups was assessed using the log rank test. In the entire group, patients with positive GSK3β expression had shorter survival times (*P* = 0.006, Figure 2A). The results also indicated that male sex, older age and squamous cell cancer (SCC) with positive GSK3β expression were associated with worse prognosis (*P* = 0.019, *P* = 0.020 and *P* = 0.002, respectively). Additionally, NSCLC patients with poorly differentiated tumors or in the relatively early stage subgroups (T1+T2, N0+N1 and M0) with positive GSK3β expression had significantly shorter survival times (*P* = 0.013, *P* = 0.003, *P* = 0.010 and *P* = 0.008, respectively; shown in Figure 2B-E).

Moreover, multivariate Cox regression analysis revealed that, in this group, tumor size, lymph node invasion and GSK3β expression were independent predictors of NSCLC prognosis. These results suggested that GSK3β plays an important role in NSCLC tumorigenesis and prompted further analysis.

### 3. GSK3β positively regulates tumor cell proliferation and survival in NSCLC

**Figure 3:**
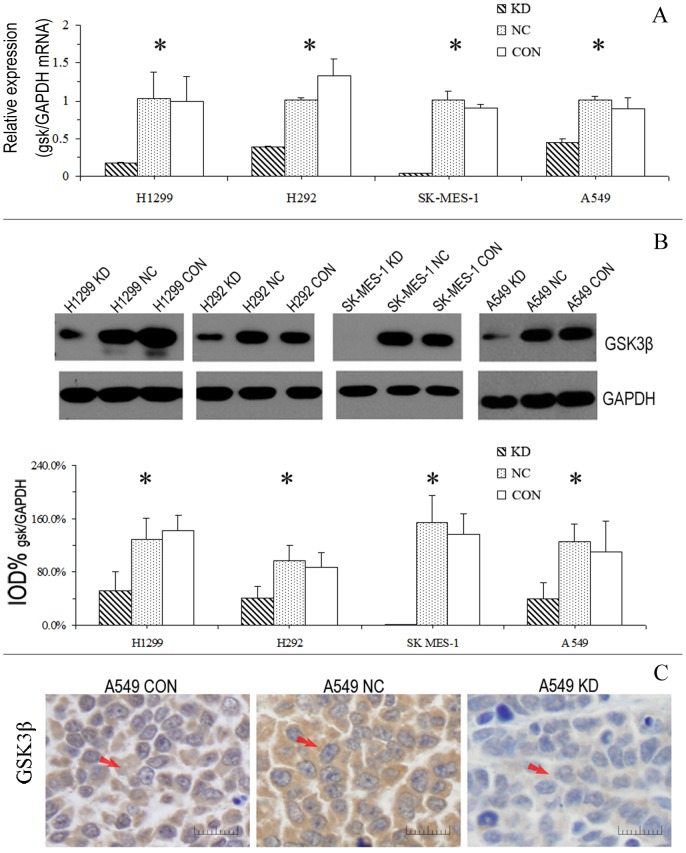
Stable silencing of GSK3β by shRNA in NSCLC cells. (**A**) GSK3β-specific shRNAs were transfected into NSCLC cells with lentiviral vector, and cells were harvested and counted 5 days after transfection. Real-time PCR indicated that interference by shRNA effectively knocked down the mRNA expression of GSK3β in NSCLC cells. (**B**) The cells were harvested and counted 7 days after transfection. Western blot shows the efficiency of protein silencing by shRNA. (**C**) After treatment with the GSK3 shRNA(KD group), the negative control shRNA (NC group) or normal saline (CON group), the A549 cells (1×10^6^/ml) were injected into the backs of nude mice. Four weeks later, xenograft tumors were isolated and immunohistochemical stained. The results verified the sustained reduced levels of GSK3β in the KD group relative to the control groups. Arrows indicate positive staining in the cytoplasm of tumor cells (magnification × 400). The experiments were repeated 3 times, and the data are expressed as the mean ± SD. *P<0.001.

A lentiviral vector was used to transfer designed shRNAs targeting GSK3β into these 4 NSCLC cell lines to persistently silence its expression. The effects of RNA interference were assessed by RT-PCR (5 days after transfection) and western blotting (7 days after transfection). These results demonstrated that the RNA and protein levels of GSK3β in tumor cells from all 4 cell lines examined were significantly reduced, as shown in Figure 3A-B (all *P*<0.001). Moreover, the xenografts tissues isolated from nude mice were immunohistochemically stained, and the results verified the sustained stable reduction in GSK3β levels after RNA interference (Figure 3C). These results indicated that GSK3β was efficiently and stably knocked down by the shRNA in all 4 NSCLC cell lines.

**Figure 4:**
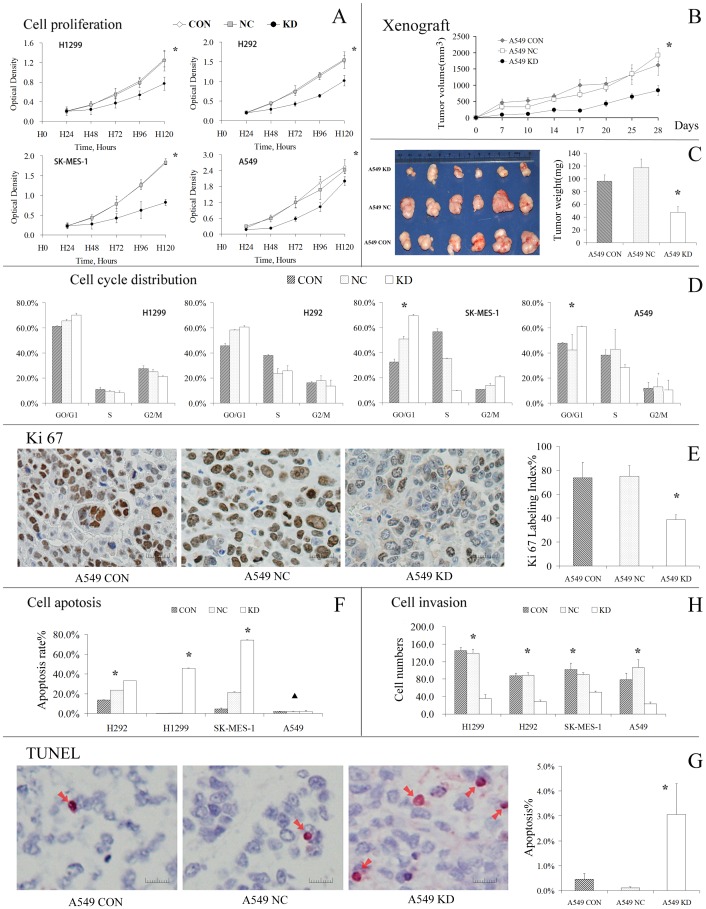
The inhibition of GSK3β attenuates cell proliferation, induces apoptosis, arrests cells in GO/G1 phase and increases cell invasiveness. (**A**) The CCK-8 test showed that the inhibition of GSK3β suppressed the proliferation of the NSCLC cell lines *in vitro*. *P<0.001. (**B**) *In vivo*: As described in Figure 3C, the xenograft models were developed in nude mice. Each group consisted of 6 animals. Tumors were observed every 3 days for 4 weeks. The xenograft tumors of mice in the KD group grew significantly slower (as determined by the tumor volume) over the entire observation period. *P<0.001. (**C**) Comparison of the isolated xenograft tumors. The image shows that tumors isolated from the KD group were smaller and lighter than the tumors from the control group. The data are expressed as the means ± SD, n = 6. *P<0.001. (**D**) Representative image of Ki-67 IHC of a tumor xenograft viewed under a microscope. To calculate the Ki-67 LI, cells with brown-stained nuclei were considered positive, indicating active proliferation (magnification × 400). The data are expressed as the means ± SD, *P<0.001. (**E**) Cell cycle analysis at 72 h after transfection. The results suggest that the inhibition of GSK3β resulted in G1/S arrest in A549 and SK-MES-1 cells and changed the proportions of the cell population in different phases of the cell cycle. The data are presented as the means ± S.D. from 3 independent experiments performed in triplicate. *P<0.001. (**F**) By flow cytometry, the apoptotic analysis results indicated that the inhibition of GSK3β greatly increased the apoptotic rate of NSCLC cells (except for A549 cells) *in vitro*. *P<0.001, ▴*P*>0.05. (**G**) The results of the TUNEL assay for xenograft tumors suggested that the inhibition of GSK3β increased the apoptotic rate of A549 cells *in vivo* (magnification × 400). The data are expressed as the means ± SD, *P<0.001. (**H**) Transwell assays showing that the inhibition of GSK3β *in vitro* significantly decreased the invasiveness of NSCLC cells. The data are expressed as the means ± S.D. *P<0.001.

The CCK-8 assay demonstrated that the knockdown of GSK3β clearly suppressed the viability of all 4 cell lines (all *P*<0.001, Figure 4A). In the *in vivo* study using xenograft mice, the tumors in the KD group grew significantly more slowly and had lower tumor volumes throughout the entire 4-week period (*P* = 0.001, Figure 4B). At the end of the observation period, the weights of the xenografts in the KD group were much lower than those for the control groups (*P* = 0.001, Figure 4C). In addition, the Ki-67 LI of the xenografts was dramatically decreased in the KD group compared with those of the NC and CON groups (38.98% vs. 75.14% vs. 73.84%, *P*<0.001, Figure 4D), which indicated that the downregulation of GSK3β inhibited cell proliferation *in vivo*. These results suggest that GSK3β positively regulates the growth and tumorigenesis of NSCLC cells, and we next sought to address how these effects were mediated.

After performing cell cycle analysis by flow cytometry, we found that inhibition of GSK3β increased the number of cells in G0/G1 phase and decreased the number of cells in S phase in A549 (*P* = 0.003, and *P*<0.001) and SK-MES-1 cells (*P* = 0.002, *P* = 0.022) (Figure 4E). In H1299 and H292 cells, GSK3β knockdown resulted in a trend toward an increase in the G0/G1 population, but the differences did not reach statistical significance (in H1299 cells, *P* = 0.056; and in H292 cells, *P* = 0.216).

*In vitro*, the cell apoptosis assay suggested that the apoptotic rates in the KD group was greatly higher than those in the control groups, with the exception of the A549 cell line (H292, H1299 and SK-MES-1, all *P<*0.001; A549, *P* = 0.461, Figure 4F). For the xenograft mice, the TUNEL assay revealed that the apoptotic rate in the KD group (3.06%±1.24%) was much higher than those of the other groups (NC group, 0.11%±0.04%; and CON group, 0.46%±0.23%) (*P*<0.001). As shown in Figure 4G, apoptotic cells were stained red and shrunken. Together, these results suggest that the inhibition of GSK3β induced NSCLC cell apoptosis.

### 4. Inhibition of GSK3β reduces the invasiveness of NSCLC cells

To count the migrated cells *in vitro*, the transwell assay using NSCLC cells was repeated 3 times, and the results are shown in Figure 4H. For all 4 cell lines, the KD group demonstrated significantly fewer invasive cells (all *P<*0.001), which suggested that the inhibition of GSK3β greatly suppressed the invasiveness of NSCLC cells. In the xenograft mice, no metastases were found upon gross inspection and palpation. In addition, light microscopy analysis revealed no evidence of metastatic foci in the fixed and stained organs.

### 5.β-catenin expression after the inhibition of GSK3β

**Figure 5:**
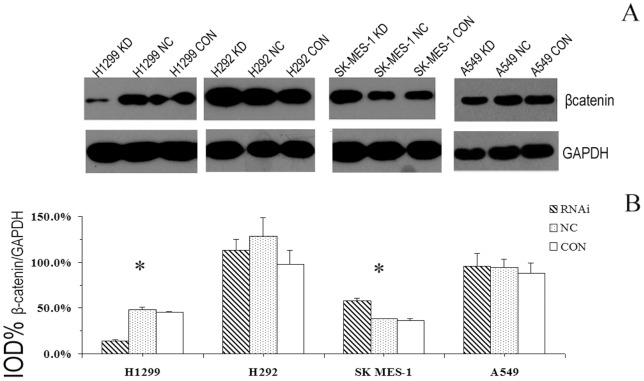
The expression of β-catenin after the inhibition of GSK3β. (**A**) Typical bands in the western blot assay showed that the protein levels β-catenin were regulated in a cell-type specific manner after the inhibition of GSK3β. (**B**) Quantitative analysis of β-catenin expression. The experiment was repeated 3 times, and the data are expressed as the means ± SD. *P<0.001.

As shown in Figure 5, the expression level of β-catenin was significantly increased in the KD group of squamous cell line SK-MES (*P*<0.001). Moreover, knockdown of GSK3β greatly suppressed the expression of β-catenin in H1299 cells (*P*<0.001) but not in H292 (*P* = 0.108) or A549 cells (*P* = 0.185).

## Discussion

GSK3β was originally identified as a major regulatory enzyme of glycogen metabolism [4], [7]. Since then, this protein has been found to be an important regulator of cell survival and apoptosis, which connects this multifunctional kinase to cancer [25], [26]. Recently, a number of studies have shown that GSK3β can positively regulate the proliferation and apoptosis of tumor cells [10]–[18], although the precise role of GSK3β in lung cancer remains unknown. Therefore, the current study explored the prognostic significance of GSK3β and its function in NSCLC.

First, the overexpression of GSK3β in NSCLC tissues was observed when comparing NSCLC tissues with adjacent normal tissues, and this aberrant accumulation of GSK3β in NSCLC cells was associated with a shorter survival time and identified as a novel risk factor for poor prognosis. Although little attention has been paid to the expression of GSK3β in lung cancer, evidence suggests that the overexpression of p-GSK3β may serve as a marker for worse prognosis in lung cancer [27]. Because p-GSK3β is the inactive form of GSK3β, overexpression of p-GSK3β is not necessarily equivalent to the loss of GSK3β, especially when the total protein level is also greatly upregulated. In our study, high expressions of pGS, which is an indicator of GSK3β activity, was detected in most of NSCLC cell lines, indicating that GSK3β is highly active in NSCLC cells. Hence, our study suggested that the overexpression of total GSK3β was over-activated and conferred poor prognosis in NSCLC patients. Similar results have been reported for bladder cancer [18]. Therefore, GSK3β overexpression may serve as a biomarker for improving early diagnosis, patient selection and the determination of prognosis.

In addtion, our IHC results showed that GSK3β is localized predominantly in the cytoplasm in patient and xenograft tumor tissues. Conversely, several studies demonstrated that GSK3β was accumulated in the nucleus in pancreatic [11], renal [28] and bladder cancers [18]. In these tumor tissues, nuclear GSK3β might play a role in NF-κB -mediated cancer cell survival; however, after knockdown of GSK3β, the NF-κB expression levels in the different NSCLC cell lines of our study were inconsistent, indicating that GSK3β may mediate cancer cell survival independent of NFκB expression (data not shown). Therefore, subcellular localization of GSK3β is complex and tightly related to its molecular mechanism. Further study on the subcellular localization role of GSK3β needs to be conducted.

The next important question is how to explain the profound influence of GSK3β on the prognosis of NSCLC patient. This study attempted to investigate the basic molecular mechanisms of GSK3β *in vitro* and *in vivo*. It has been well established that GSK3β plays a central role in the regulation of apoptosis [26]. For example, GSK3β knockout or lithium treatment potentiates apoptosis in hepatocytes [29], and an increased rate of apoptosis following the inhibition of GSK3β has been reported in prostate cancer [30], pancreatic cancer [11], leukemia [13], glioma [17] and bladder cancer [18]. Furthermore, it has been suggested that GSK3β can inhibit cell death through the death receptor-mediated extrinsic apoptotic signaling pathway [26]. The results of our *in vitro* experiments revealed clear apoptotic abnormalities after GSK3β inhibition in the examined cell lines, with the exception of A549 cells. In addition, the inhibition of GSK3β in A549 cells induced significant apoptosis in the xenograft tumors of nude mice. Therefore, the results of previous studies and our current study suggest that GSK3β has anti-apoptotic effects in NSCLC, which cause it to function as a tumor promoter in lung cancer.

GSK3β is centrally involved in diverse cellular processes, including cell cycle regulation [5]–[7]. In this study, the intrinsic inhibition of GSK3β arrested NSCLC tumor cells in the G0/G1 phase *in vitro*. In xenografted mice, the clearly reduced Ki-67 LI after GSK3β knockdown indicated that a greater proportion of cells were in a resting state (G0). These findings suggest that GSK3β positively regulates the cell cycle and subsequently promotes tumor cell proliferation. Cao *et al*. demonstrated that the overexpression of GSK3β increased cyclin D1 activity, induced entry from G0/G1 into S phase and facilitated the proliferation of ovarian cancer cells [14]. In prostate cancer, it was also found that lithium suppressed cell proliferation by disrupting the E2F-DNA interaction and reducing S-phase gene expression [31]. Similar results have been reported for hepatocellular carcinoma cells [32], and these findings are in accordance with our results.

The role of GSK3β in cancer cell motility has not been thoroughly studied, and the current results obtained for different types of cancers are conflicting. The selective GSK3β inhibitor lithium has been reported to promote the motility of colon cancer cells [33], whereas treating cultured cells with GSK3 inhibitors or small interfering RNA (siRNA) targeting GSK3 has been reported to inhibit motility [34]. In addition, lithium can reduce the invasiveness of glioma cells, and this effect may be related to GSK3β [35]. Moreover, many GSK3 substrates are known to be involved in cell migration [36], which increases the complexity of this situation. In this study, it was demonstrated that the inhibition of GSK3β greatly suppressed the invasiveness of NSCLC cells, regardless of the cancer type, and this result supports the prognostic value and tumor promoter role of GSK3β in lung cancer.

The results of our study clearly indicate that GSK3β positively regulates cell proliferation, tumorigenesis, apoptosis and cell invasiveness in NSCLC, which explains the prognostic value of this protein. Because GSK3β has many protein targets, it was not clear which downstream targets of GSK3 mediated these effects on cell survival and invasion. Therefore, we investigated the changes in the levels of β-catenin, a well-known substrate of GSK3β, after the knockdown of GSK3β using siRNA.

Theoretically, the inactivation of GSK3β can lead to increased cytoplasmic β-catenin levels and the activation of the Wnt signaling pathway [25], [26]. However, our results demonstrated that after inhibition of GSK3β, overactive β-catenin was only present in SK-MES-1 cells. This finding conflicts with the result that GSK3β overexpression in squamous cell cancer is associated with worse prognosis. In addition, after knockout of GSK3β, the protein level of β-catenin was inconsistent in the lung adenocarcinoma cells, suggesting that in NSCLC cells, the expressions of β-catenin is regulated in a cell-type specific manner. GSK3β seems to function independent of the β-catenin pathway in lung cancer, and recent studies of gastric [37], colorectal [12] and hepatic [32] cancers have yielded similar results.

In summary, this study demonstrated that GSK3β is deregulated in NSCLC and that GSK3β overexpression serves as a powerful, independent negative prognostic marker. Moreover, the inhibition of GSK3β activity reduced cell survival and proliferation, induced apoptosis and suppressed tumor invasiveness. This evidence indicates that GSK3β plays a crucial role in the development of NSCLC. Moreover, these results could not be attributed to β-catenin; therefore further research into the intrinsic mechanism responsible for these effects is necessary.
